# Differential Adaptation of *Candida albicans* In Vivo Modulates Immune Recognition by Dectin-1

**DOI:** 10.1371/journal.ppat.1003315

**Published:** 2013-04-18

**Authors:** Mohlopheni J. Marakalala, Simon Vautier, Joanna Potrykus, Louise A. Walker, Kelly M. Shepardson, Alex Hopke, Hector M. Mora-Montes, Ann Kerrigan, Mihai G. Netea, Graeme I. Murray, Donna M. MacCallum, Robert Wheeler, Carol A. Munro, Neil A. R. Gow, Robert A. Cramer, Alistair J. P. Brown, Gordon D. Brown

**Affiliations:** 1 Division of Immunology, Institute of Infectious Disease and Molecular Medicine, University of Cape Town, Observatory, Cape Town, South Africa; 2 Aberdeen Fungal Group, University of Aberdeen, Institute of Medical Sciences, Foresterhill, Aberdeen, United Kingdom; 3 Department of Microbiology and Immunology, Geisel School of Medicine at Dartmouth, Hanover, New Hampshire, United States of America; 4 Molecular and Biomedical Sciences, University of Maine, Orono, Maine, United States of America; 5 Nijmegen Institute for Infection, Inflammation and Immunity (N4i), and University Medical Centre Nijmegen, Nijmegen, The Netherlands; 6 Pathology, Division of Applied Medicine, University of Aberdeen, Foresterhill, Aberdeen, United Kingdom; University of Wisconsin-Madison, United States of America

## Abstract

The β-glucan receptor Dectin-1 is a member of the C-type lectin family and functions as an innate pattern recognition receptor in antifungal immunity. In both mouse and man, Dectin-1 has been found to play an essential role in controlling infections with *Candida albicans*, a normally commensal fungus in man which can cause superficial mucocutaneous infections as well as life-threatening invasive diseases. Here, using *in vivo* models of infection, we show that the requirement for Dectin-1 in the control of systemic *Candida albicans* infections is fungal strain-specific; a phenotype that only becomes apparent during infection and cannot be recapitulated *in vitro*. Transcript analysis revealed that this differential requirement for Dectin-1 is due to variable adaptation of *C. albicans* strains *in vivo*, and that this results in substantial differences in the composition and nature of their cell walls. In particular, we established that differences in the levels of cell-wall chitin influence the role of Dectin-1, and that these effects can be modulated by antifungal drug treatment. Our results therefore provide substantial new insights into the interaction between *C. albicans* and the immune system and have significant implications for our understanding of susceptibility and treatment of human infections with this pathogen.

## Author Summary

Dectin-1 is a pattern recognition receptor recognising the fungal cell-wall component, β-glucan, and plays an essential role in controlling *C. albicans* infections in both mouse and man. *Candida albicans* is part of the normal human microflora, yet is capable of causing superficial mucosal infections as well as life-threatening invasive diseases, particularly in patients whose immune function is compromised. Here we found that the contribution of Dectin-1 is limited to specific strains of *C. albicans*; effects which are due to the differential adaptation of these pathogens during infection. Importantly, *C. albicans* strains showed variations in both the composition and nature of their cell walls, and it was these differences which influenced the role of Dectin-1. Crucially, we found that we could alter the fungal cell wall, and subsequent interactions with the host, using antifungal drugs. These findings have substantial implications for our understanding of the factors contributing to human susceptibility to infections with *C. albicans*, but also treatment strategies.

## Introduction

The immune system of healthy individuals has effective mechanisms for preventing fungal infection, yet immunosuppressive infections, such as HIV/AIDS, and modern immunosuppressive and invasive medical interventions can substantially increase the risk of infection with numerous fungal pathogens. *Candida albicans* is one such microorganism, which is normally found as a commensal on host epithelial surfaces, colonizing more than 50% of individuals [1]. However, in severely immunocompromised patients, those enduring invasive clinical procedures or those requiring extended stays in intensive care units, *Candida* species are the most important etiological agent of life-threatening, invasive systemic and bloodstream fungal infections [1].

In addition to life-threatening invasive infections, superficial mucocutaneous infections with *Candida* species are also common, even in immuno-competent individuals. While the underlying mechanisms are still incompletely understood, a link to defects in Th17 immunity have been identified in some patients [2]. Indeed, mutations in STAT1, STAT3, IL-17 and IL17RA all result in susceptibility, especially to mucocutaneous candidiasis [2]. Furthermore, C-type lectin receptors (CLR) and their intracellular signalling pathways, particularly the CARD9 pathway, are now recognized to play a predominant role in driving these and other protective antifungal immune responses [3]. CLRs are required for the recognition and ingestion of fungi by phagocytes, the induction of antimicrobial effector mechanisms and inflammatory mediators, and they direct and modulate adaptive immunity, including Th17 responses [3].

One such CLR is the β-glucan receptor, Dectin-1, which can mediate multiple cellular functions through its cytoplasmic signalling domain including phagocytosis, the respiratory burst, and the production of soluble factors, such as cytokines, chemokines and eicosanoids [3]. The importance of Dectin-1 has been exemplified in our recent studies that have implicated it in the control of antifungal immunity in humans [4], [5]. An essential role for Dectin-1 has also been demonstrated in mouse models by several groups, where loss of Dectin-1 resulted in a failure to mount protective inflammatory responses and an inability to control fungal growth [6], [7], [8], [9], [10]. During infection with *C. albicans*, these defects resulted in systemic and mucosal susceptibility [6], [7], [8], [9]. Surprisingly another group found no role for Dectin-1 in immunity to systemic infections with this pathogen, however, this study was performed using a different strain of *C. albicans* and a different mouse genetic background [11]. As innate recognition of *C. albicans* can be influenced by both fungal and mouse strain [9], [12], [13], [14], we have explored the contribution of these variables to Dectin-1-mediated recognition of this pathogen.

## Results

### Dectin-1 dependency is *Candida albicans* strain specific

**Figure 1:**
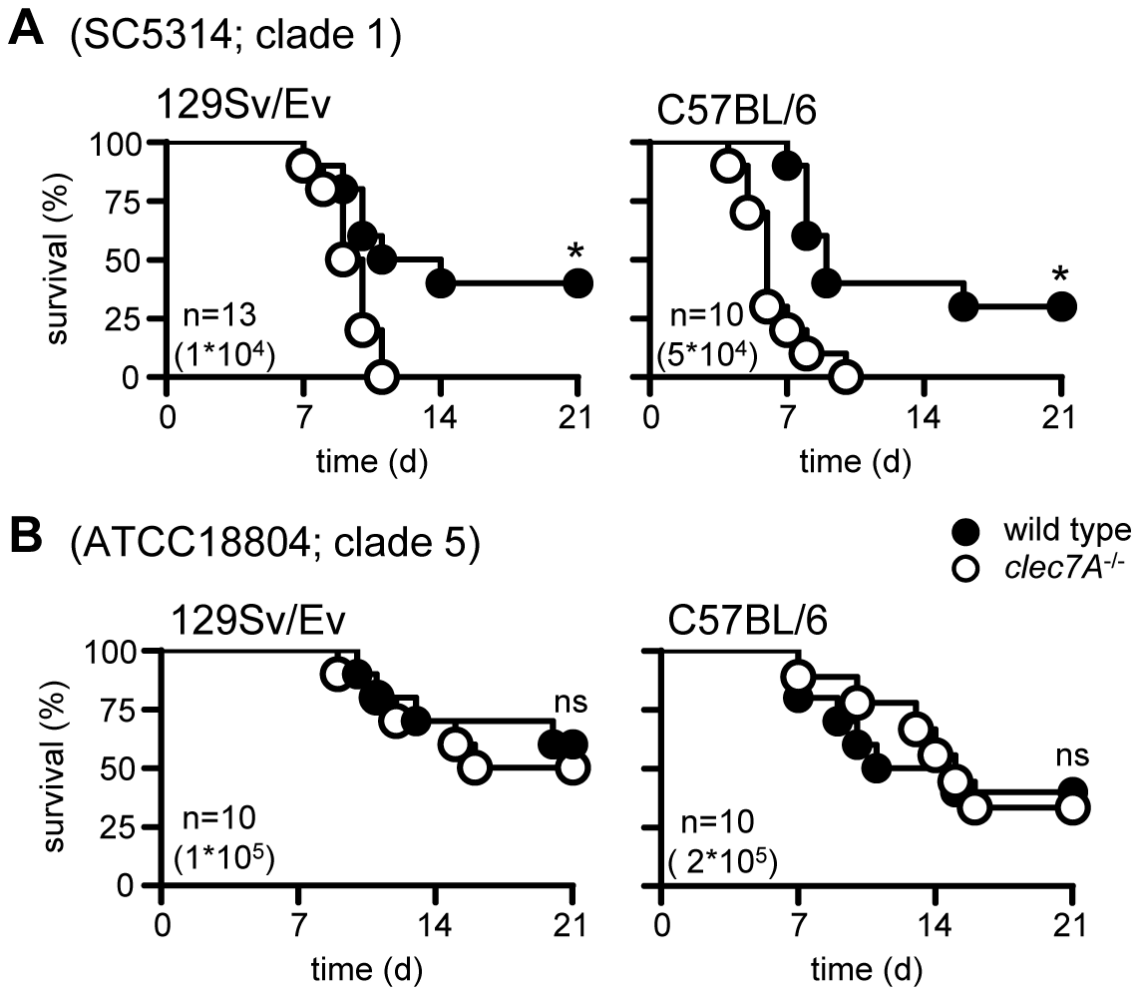
Dectin-1 dependence is related to fungal strain and not mouse background. Survival analysis of 129/Sv and C57BL/6 wild-type and Dectin-1*^−/−^* mice following infection with (**A**) *C. albicans* SC5314 or (**B**) ATCC18804. The data shown are representative of at least two independent experiments. Also shown are number of animals per group (n) and dosage used for infection. *; p<0.05. See also Table S1.

Using 129Sv mice, we had found that Dectin-1 was essential for controlling systemic infection with *C. albicans* strain SC5314 [6]. In contrast, Iwakura's group had found that Dectin-1 was not required for the control of this pathogen; their experiments being performed using C57BL/6 mice and the *C. albicans* strain ATCC18804 [11]. To explore the contribution of mouse and/or fungal strain to the Dectin-1-mediated recognition of this pathogen, we backcrossed our 129/Sv background knockout mice for nine generations onto the C57BL/6 background (data not shown), and then assessed the role of Dectin-1 in both mouse backgrounds with both strains of *C. albicans*. Consistent with our earlier results [6], Dectin-1^−/−^ mice (*clec7A^−/−^*) on a 129/Sv background showed significantly enhanced susceptibility to infection with *C. albicans* SC5314 (Figure 1A). Moreover, similarly enhanced susceptibility to this strain of *C. albicans* was also observed in the knockout mice on a C57BL/6 background. In contrast, loss of Dectin-1 had no effect on susceptibility to *C. albicans* ATCC18804 on either murine background (Figure 1B). Thus Dectin-1-mediated control of systemic *C. albicans* infection is not dependent on the genetic background of the mice.

**Figure 2:**
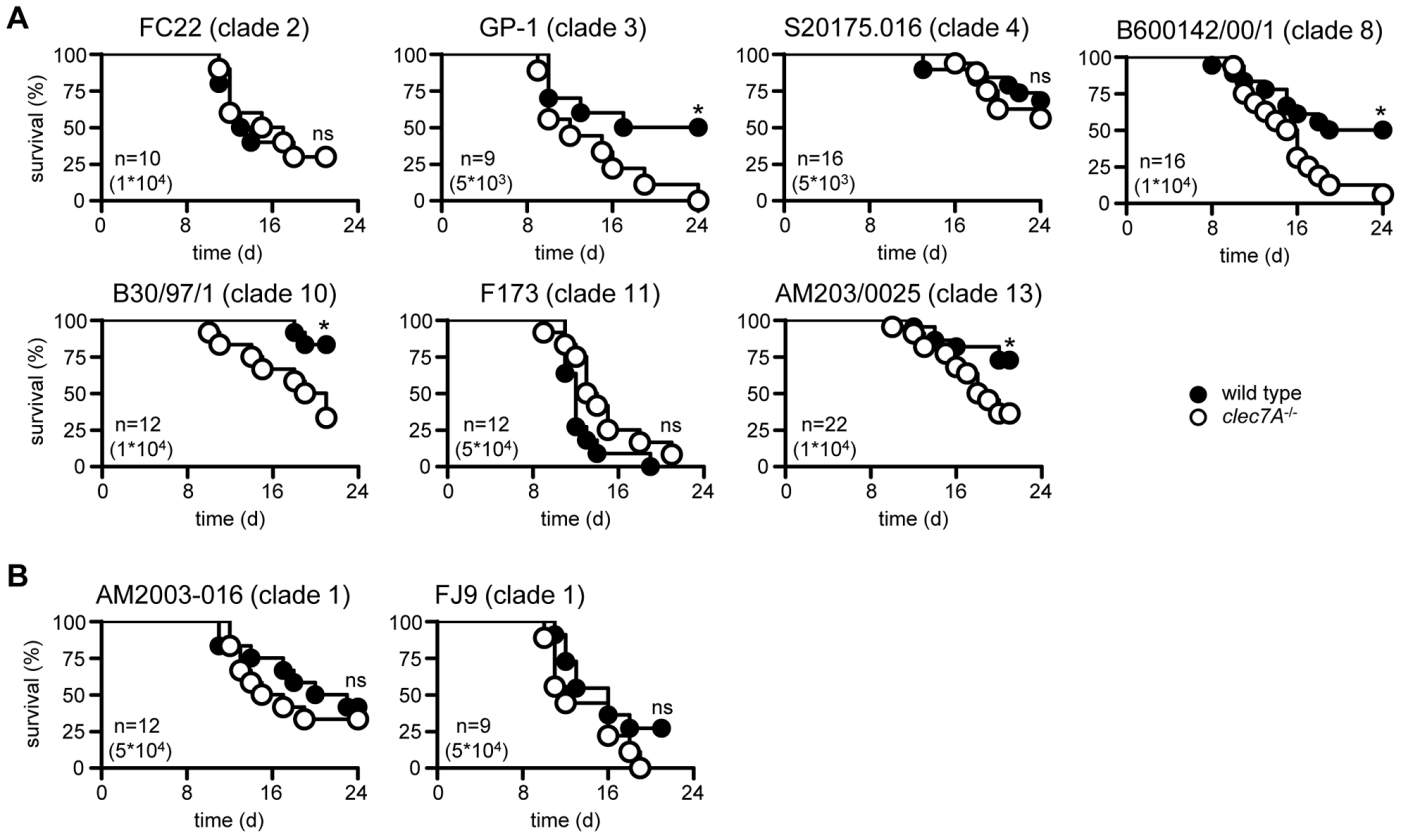
Dectin-1 dependence is not related to the clade of *C. albicans*. Survival analysis of 129/Sv wild-type and Dectin-1*^−/−^* mice following infection with strains from (**A**) several different clades of *C. albicans*, or (**B**) two additional isolates from clade 1 as indicated. The data shown are representative of at least two independent experiments. Also shown are number of animals per group (n) and dosage used for infection. *; p<0.05. See also Table S1.

Strains of *C. albicans* have been subdivided into 17 clades based on multilocus sequence typing [15] and as the two strains used in these experiments (SC5314 and ATCC18804) come from different clades (1 and 5, respectively), we explored the possibility that Dectin-1 dependency is associated with different clades of *C. albicans*. Wild type and Dectin-1^−/−^ mice were infected with representative isolates from several clades (listed in Table S1), as well as two additional isolates from clade 1 (i.e. the same clade as SC5314), and survival of these animals was assessed over time. Dectin-1 was required to control infection with roughly half of the strains tested (Figure 2A), as evidenced by significantly increased mortality in the knockout mice. However, this receptor was not required to control infection with other strains from clade 1 (Figure 2B). Thus we conclude that Dectin-1 dependency is *C. albicans* strain-specific, but does not correlate with particular clades of this pathogen.

### Strain-specific Dectin-1-dependency corresponds with enhanced fungal burdens and dysregulated cytokine responses during systemic infection

**Figure 3:**
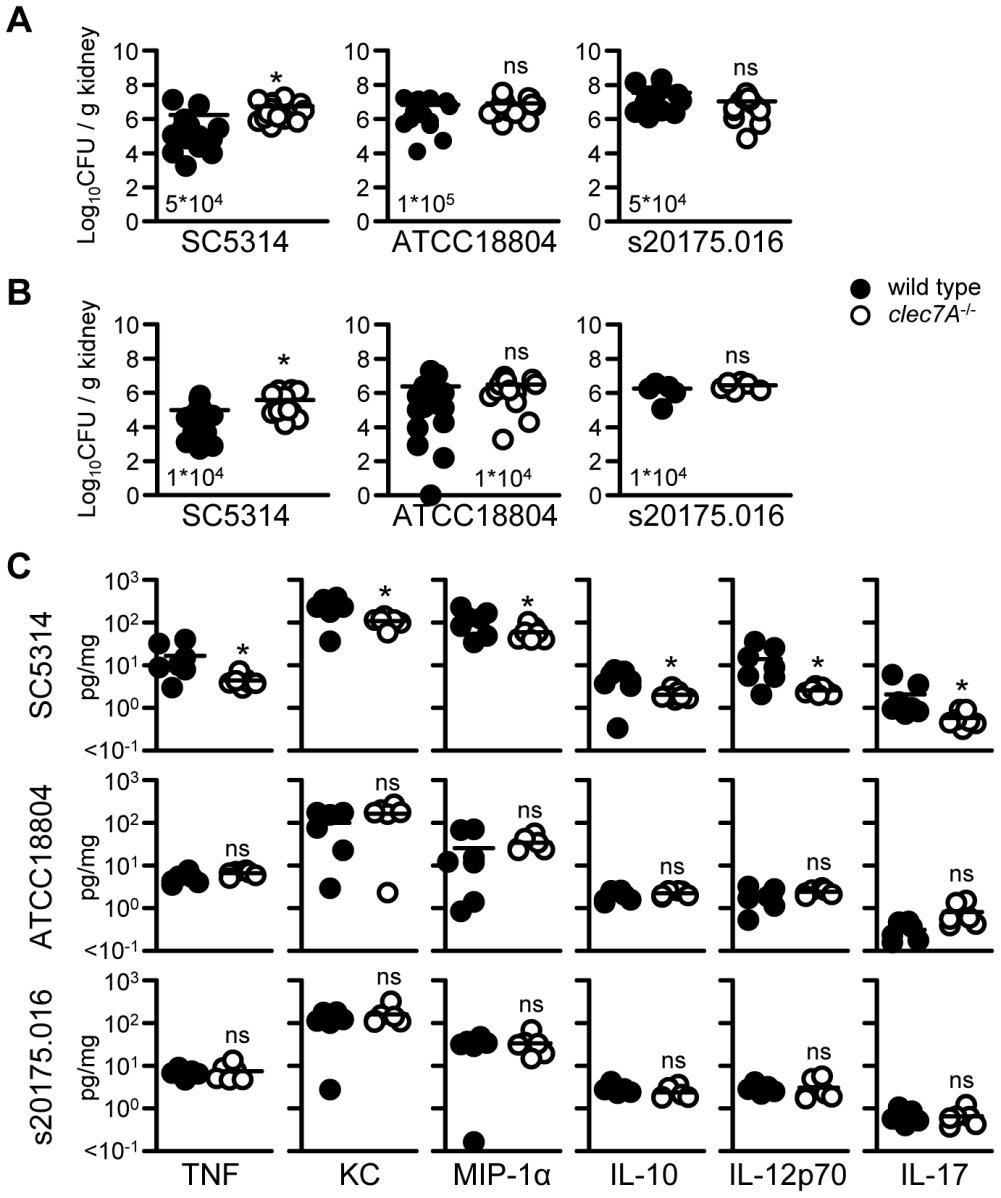
Loss of Dectin-1 is associated with increased fungal burdens and dysregulated cytokine responses with specific strains of *C. albicans*. Fungal burdens in the kidneys of 129/Sv wild-type and Dectin-1*^−/−^* mice at day 9 post-infection with a (**A**) high or a (**B**) low dose of various *C. albicans* strains, as indicated. (**C**) Characterisation of cytokine levels in the kidneys of 129/Sv wild-type and Dectin-1*^−/−^* mice at day 9 post-infection with a high dose (as in **A**) of various *C. albicans* strains, as indicated. Shown are data from two pooled (**A**, **B**) and one representative (**C**) experiment. Each experiment involved 7–10 mice per group. Bar indicates the mean.*; p<0.05.

To gain further insight into the mechanisms underlying the difference in Dectin-1 dependency between *C. albicans* SC5314 and ATCC18804, we examined fungal burdens and cytokine responses in the kidneys of infected wild-type and Dectin-1^−/−^ mice. These analyses were performed on day 9, a time point chosen when the animals were just starting to succumb to the infection (see Figure 1). In line with our survival analysis, we observed significantly higher fungal burdens in the Dectin-1^−/−^ animals infected with *C. albicans* SC5314, using both high-dose (Figure 3A) and low-dose inocula (Figure 3B). Moreover, these mice also had substantially altered levels of cytokines known to be critical for controlling *C. albicans* infection, as we reported previously [6] (Figure 3C). In contrast, loss of Dectin-1 had no effect on fungal burdens or cytokine responses in mice infected with *C. albicans* ATCC18804 (Figure 3A–C).

We also assessed the possibility that these *in vivo* differences may be related to the virulence of the individual strains. Indeed, a 4- to 10-fold higher inoculum of ATCC18804 was required to induce a level of mortality in mice that was roughly equivalent to that of SC5314 (Figure 1 and Table S1). We therefore examined kidney fungal burdens and cytokine responses with the Dectin-1-independent s20175.016 *C. albicans* strain, which was roughly equivalent in virulence to SC5314 (Figure 2 and Table S1). However, as we observed for ATCC18804, neither fungal burdens nor cytokines responses were affected by Dectin-1 deficiency during infection with s20175.016 (Figure 3A–C). Taken together, these results demonstrate that the absence of Dectin-1 results in substantially increased fungal burdens, dysregulated cytokine responses and enhanced susceptibility to infection, but only with specific strains of *C. albicans*.

### Dectin-1 dependency cannot be recapitulated *in vitro*

**Figure 4:**
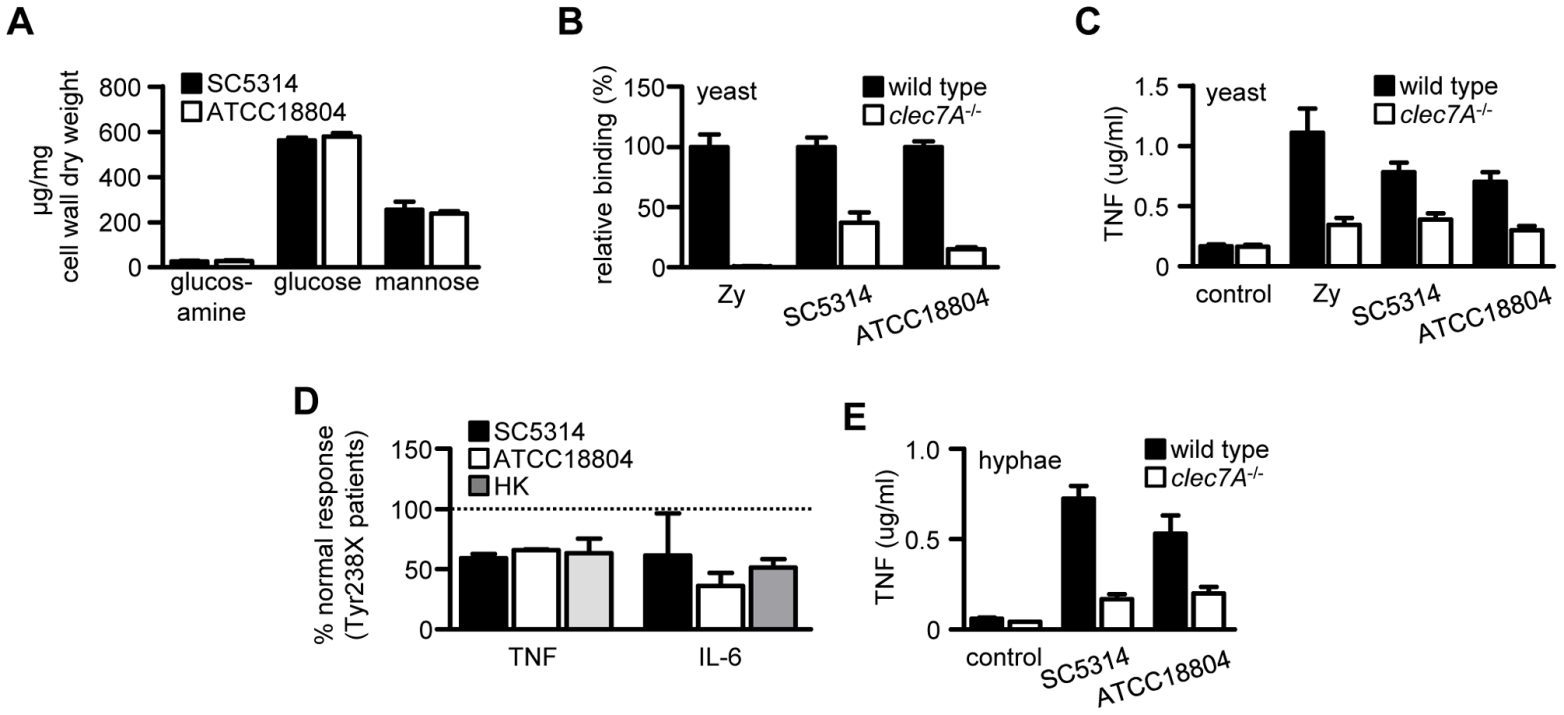
The dependence of Dectin-1 cannot be recapitulated *in vitro.* (**A**) Cell wall biochemical composition of *in vitro* grown *C. albicans* SC5314 and ATCC18804 yeast cells, as indicated. (**B**) Relative binding of fluorescently-labelled live *C. albicans* yeast cells or zymosan (Zy) to C57BL/6 wild-type and *Clec7A^−/−^* thioglycollate-elicited peritoneal macrophages, as indicated. (**C**) Measurement of TNF in culture supernatants from C57BL/6 wild type versus *Clec7A^−/−^* peritoneal macrophages after stimulation with live *C. albicans* yeast cells or zymosan (Zy), as indicated. (**D**) Measurement of TNF and IL-6 responses from homozygous Y238X patients after stimulation with *C. albicans* yeast cells or heat-killed yeast (HK; strain ATCC MYA-3573), as indicated. Results were normalized to the treated cells from normal individuals. (**E**) Measurement of TNF responses from 129S/Sv wild type versus *Clec7A^−/−^* peritoneal macrophages after stimulation with live *C. albicans* hyphae, as indicated. Data shown are means ± SEM of pooled data from at least two independent experiments, except for (**E**), which is the mean ± SD of a representative experiment.

Given the clear-cut difference in Dectin-1 dependency between infections with SC5314 and ATCC18804 *in vivo*, we reasoned that there might be substantial differences in the cell wall β-glucan content of these fungal strains. However, the cell walls of these strains grown *in vitro* revealed nearly identical levels of glucosamine, glucose and mannose, reflecting equivalent amounts of chitin, β-glucan and mannan, respectively (Figure 4A). To confirm this crude biochemical analysis and verify that the cell walls of SC5314 and ATCC18804 were also similar from an immunological perspective, we compared recognition of these fungal strains using wild type and Dectin-1^−/−^ thioglycollate-elicited macrophages *in vitro*. Using fluorescently labelled live yeast cells, and zymosan particles as a positive control [6], we demonstrated that they bound to macrophages at equivalent levels. Importantly, the binding of both strains and zymosan was Dectin-1 dependent (Figure 4B). Consistent with these observations, both strains induced inflammatory cytokine responses, determined by measuring TNF, to similar levels and in a Dectin-1 dependent manner (Figure 4C). Moreover, we demonstrated the Dectin-1 dependent nature of recognition of these strains using human monocytes deficient in Dectin-1, isolated from individuals homozygous for the Y238X polymorphism of the receptor [5] (Figure 4D). Thus the yeast forms of these strains demonstrate an equivalent requirement for Dectin-1 recognition *in vitro.*

**Figure 5:**
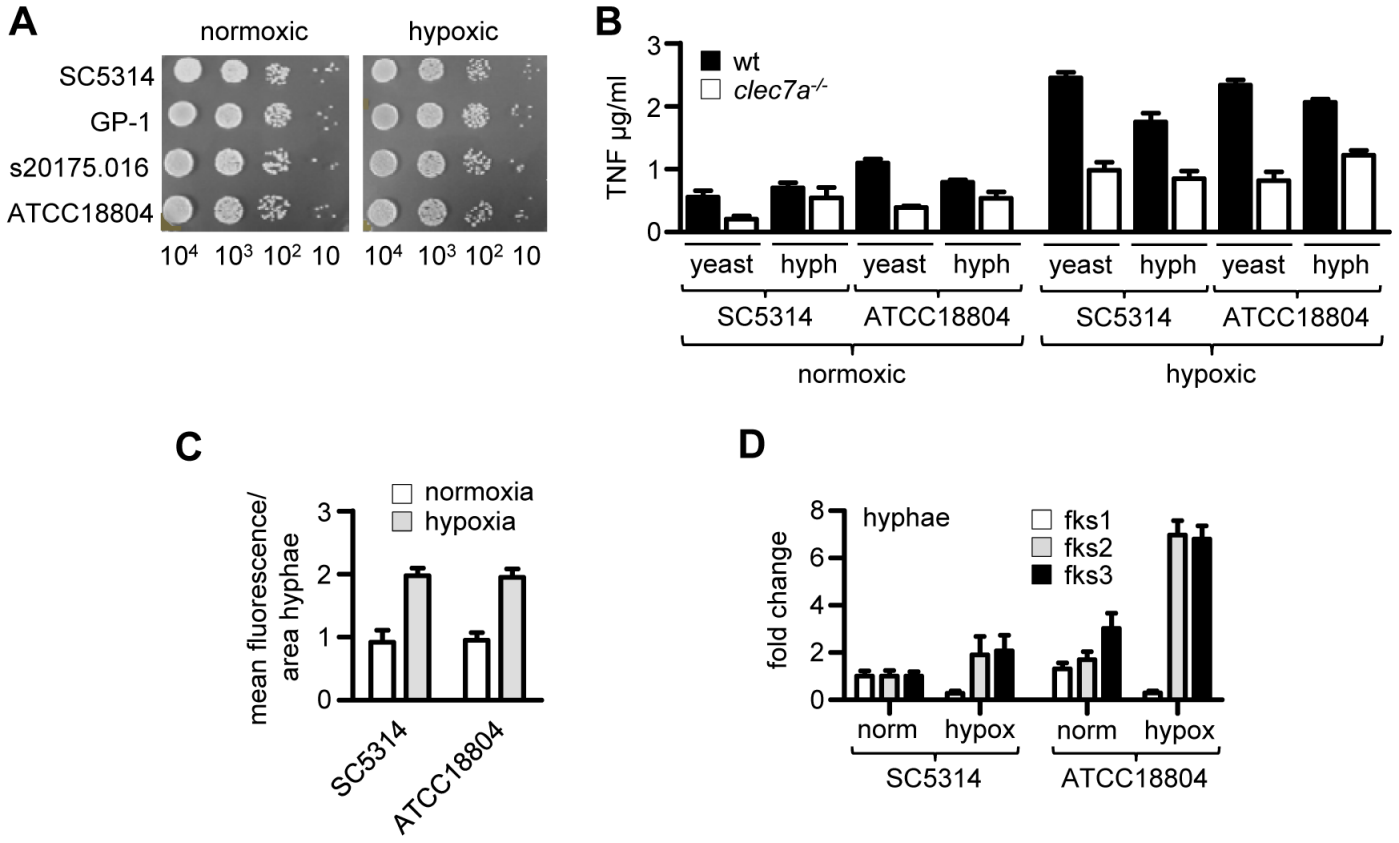
Dectin-1-responsiveness but not dependence is induced by hypoxia *in vitro*. (**A**) Growth of various strains of *C. albicans* under normoxia or hypoxia, as indicated. (**B**) Measurement of TNF responses from C57BL/6 wild type versus *Clec7A^−/−^* peritoneal macrophages after stimulation with UV-irradiated *C. albicans* yeast or hyphae, grown under normoxic or hypoxic conditions, as indicated. (**C**) Quantitation of β-glucan exposure on UV-irradiated *C. albicans* hyphae, grown *in vitro* under conditions of normoxia or hypoxia, as indicated, and stained with soluble Dectin-1. (**D**) Fold change in *FKS* gene expression in C. albicans hyphae under normoxic versus hypoxic conditions, as indicated. Data shown (mean ± SD) are from one representative experiment.

The exposure of β-glucans on *C. albicans*, and hence recognition by Dectin-1, may be restricted to the yeast form of this fungus [16], although this is controversial [17]. Nevertheless, we explored the possibility that the strain-linked dependence for Dectin-1 may stem from differential recognition of fungal hyphae, particularly as this morphological form is abundant in kidney lesions *in vivo* [6]. However, as we found for yeast cells, co-culture of thioglycollate-elicited macrophages with pre-formed live *C. albicans* hyphae of both strains induced similar levels of TNF (Figure 4E). Importantly, these responses were equivalently Dectin-1 dependent. Similar results were also obtained when the hyphae were killed using UV-irradiation (see Figure 5B). Thus the exposure of β-glucans on *in vitro* generated hyphae, and subsequent recognition by Dectin-1, does not differ between the two strains of *C. albicans*.

We then considered the possibility that differential exposure of β-glucans by these *C. albicans* strains may be occurring under conditions of hypoxia, mimicking the conditions *in vivo* and which we have shown to alter cell wall β-glucan content and immune recognition of *Aspergillus* [18]. We therefore first examined the growth of several *C. albicans* strains from different clades and observed no obvious effects of hypoxia on the growth of these organisms, when compared to cells grown under normoxic conditions (Figure 5A). Subsequently, we examined the inflammatory response of wild type and Dectin-1^−/−^ thioglycollate-elicited macrophages to UV-irradiated SC5314 and ATCC18804 yeast cells and hyphae, which were grown under normoxic or hypoxic conditions (Figure 5B). In all cases, there were no substantial differences in TNF production between SC5314 and ATCC18804, and these responses all required Dectin-1. Interestingly, yeast and hyphal cells from both strains that were grown under hypoxia were more inflammatory, inducing much higher levels of TNF and displaying a greater Dectin-1 dependency (Figure 5B). Consistent with these observations, when grown under hypoxia, we detected higher levels of exposed β-glucans on cells from both strains under these conditions (Figure 5C), which correlated with an increased expression of genes involved in β-glucan synthesis (Figure 5D). Thus, we conclude that the strain dependency for Dectin-1 is not recapitulated *in vitro*, at least using the parameters examined here.

### Dectin-1 dependency is not related to the level of fungal β-glucan exposure *in vivo*

**Figure 6:**
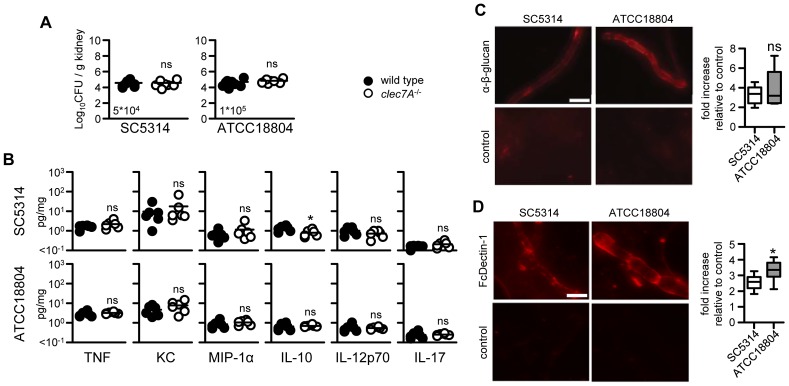
Dectin-1 dependence is not related to β-glucan exposure *in vivo.* (**A**) Fungal burdens in the kidneys of 129/Sv wild-type and Dectin-1*^−/−^* mice at day 3 post-infection with various *C. albicans* strains, as indicated. (**B**) Characterisation of cytokine levels in the kidneys of 129/Sv wild-type and Dectin-1*^−/−^* mice at day 3 post-infection of various *C. albicans* strains, as indicated. Images and quantitation of β-glucan expression on fungal cells isolated from the infected kidneys of C57BL/6 mice at day 9 and stained with (**C**) anti-β-glucan antibodies or (**D**) soluble Dectin-1 (FcDectin-1). Control cells were stained with secondary antibody only. Data shown are from a representative experiment. Bar indicates the mean.*; p<0.05. ns, not significant.

Following infection of mice, *C. albicans* cell-wall β-glucans are thought to be initially masked and only become exposed after several days [17]. Indeed, early during infection, at day 3, we observed that fungal burdens (Figure 6A) and cytokine responses (Figure 6B) were not influenced by the loss of Dectin-1, irrespective of the *C. albicans* strain. This suggested that the fungal-strain dependency for Dectin-1 could be related to differential unmasking of β-glucans at later time points *in vivo*. To explore this, we examined the exposure of β-glucans *in vivo* at day 9 after infection, a time point when these carbohydrates become exposed [17], and where we had observed significant differences in fungal burdens and cytokine responses (see Figure 2). Surprisingly, when staining with an anti-β-glucan antibody, we observed equivalent exposure of β-glucans in both strains (Figure 6C). To verify that this result reflected immunological recognition by Dectin-1, we also examined β-glucan exposure using a soluble version of this CLR as a probe (Figure 6D). In fact, using this approach, we found that the ATCC18804 strain exposed significantly more β-glucan than SC5314. Thus, these results paradoxically suggest that the strain-linked Dectin-1 dependence observed *in vivo* does not correlate with the level of exposed β-glucan.

### Dectin-1-mediated recognition is linked to cell wall composition and architecture *in vivo*

**Figure 7:**
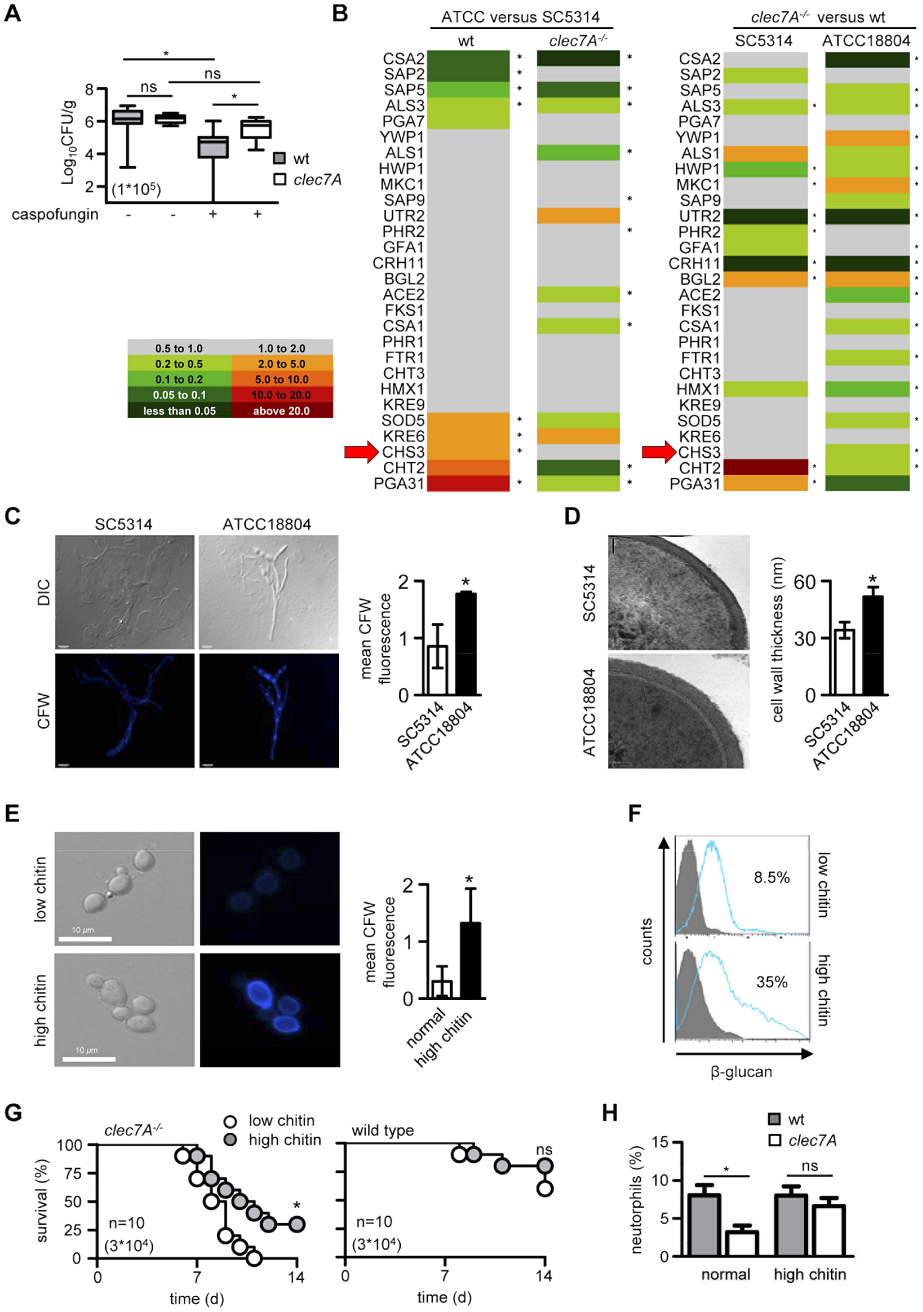
Dectin-1 dependence *in vivo* is related to changes in the fungal cell wall and chitin content. (**A**) Box and whisker graph showing fungal burdens in the kidneys of 129/Sv wild-type and Dectin-1*^−/−^* mice at day 7 post-infection with ATCC18804, with and without caspofungin treatment, as indicated. (**B**) Comparative expression analysis of genes encoding selected cell wall associated and secreted proteins, as indicated, on fungal mRNA isolated from the kidneys of 129/Sv mice infected with SC5314 or ATCC18804 at day 7. See also Table S2. (**C**) Confocal images and quantitation of chitin levels in fungal cells isolated from the kidneys of 129/Sv mice infected with SC5314 and ATCC18804 at day 7, as indicated. (**D**) TEM images (left) and quantification of cell wall thickness (right) of fungal cells isolated from the kidneys of 129/Sv mice infected with SC5314 and ATCC18804 at day 7. Scale bar = 50 nm. (**E**) Confocal images and quantitation of chitin levels of *in vitro* cultured normal and high chitin SC5314 fungal cells, as indicated. Scale bar = 10 µm. (**F**) Flow cytometric analysis of exposed β-glucan on low and high-chitin containing *in vitro* grown *C. albicans* SC5314, using soluble Dectin-1 as a probe. The filled histograms represent secondary only control and the blue histogram indicates FcDectin-1 staining. (**G**) Survival analysis of 129/sv Dectin-1*^−/−^* or wt mice following infection with 3×10^4^ CFU high-chitin or normal-chitin containing *C. albicans* SC5314. (**H**) Intraperitoneal inflammation, as measured by neutrophil influx, 4 hr after i.p. infection with 1×10^5^ CFU high-chitin or normal-chitin containing *C. albicans* SC5314 in Dectin-1*^−/−^* or wt mice, as indicated. All data shown are from a representative experiment, except for (H) which is pooled data from two experiments. Bar indicates the mean.*; *p*<0.05. ns, not significant.

Interactions of *C. albicans* with the innate immune system involve many different fungal cell wall pathogen associated molecular patterns (PAMPs) [19]. We therefore tested whether induced modifications in the cell wall structure, and hence PAMP exposure, would alter innate recognition of the ATCC18804 strain. For these experiments we examined the effects of caspofungin, an echinocandin antifungal drug whose actions significantly change cell wall architecture and have previously been shown to influence β-glucan exposure [17], [20]. As before, equivalent fungal burdens were observed in the kidneys of wild type and Dectin-1^−/−^ mice infected with ATCC18804 (Figure 7A). However, while treatment with caspofungin significantly reduced fungal burdens in the kidneys of wild-type mice, it had no effect on colonization in the kidneys of the Dectin-1^−/−^ animals; i.e. the clearance of this organism following treatment with caspofungin was now Dectin-1-dependent (Figure 7A). Thus changes in cell wall architecture can alter the dependency on Dectin-1, following infection with an otherwise Dectin-1-independent strain of *C. albicans.*

To gain insight into the cell wall components that contribute to the Dectin-1-independence of ATCC18804, we compared the expression profiles of a range of selected genes in both ATCC18804 and SC5314 cells isolated from the kidneys of infected wild-type and Dectin-1-knockout mice. The genes examined encoded many cell wall-associated and secreted functions, including genes involved in chitin and β-glucan biosynthesis, cell wall remodelling, adhesion, secreted proteases, iron assimilation, and transcription factors that regulate cell wall biogenesis (Table S2). We found that the expression of many of these genes did not differ significantly between these strains, or during infection in wild-type versus Dectin-1^−/−^ mice. However, several cell-wall associated genes were differentially expressed under these conditions. Interestingly, the ATCC18804 strain appeared more adaptable to the immunological status of the host, as suggested by the relatively large subset of ATCC18804 genes (60%) that displayed significant differences in expression in the wild-type versus the Dectin-1^−/−^ animals (Figure 7B, right panel). Notably, the virulence related genes *ALS3*, *CSA2* and *SAP5* were expressed at lower levels in ATCC18804 versus SC5314, in both wild-type and Dectin-1^−/−^ animals, possibly reflecting the differences in virulence of these strains (Figure 7B and see Figure 1).

Of particular interest was the chitin synthase gene *CHS3* (Figure 7B, red arrow), which was strongly and reproducibly up-regulated in ATCC18804, versus SC5314, but only in wild type mice. Furthermore, two other genes implicated in chitin regulation (*PGA31* and *CHT2*, [21]) also displayed significant differential expression between strains. This suggested that there may be differences in chitin levels in ATCC18804 and SC5314 *in vivo*. To explore this possibility, we determined the levels of this cell-wall component in fungal cells isolated directly from infected mouse kidneys using Calcofluor White. Consistent with the increased expression of these chitin-related genes described above, we detected significantly elevated chitin levels in ATCC18804 cells compared to SC5314 cells (Figure 7C). Moreover, TEM analysis revealed substantial differences in the architecture of their cell walls (Figure 7D).

These data indicate that the Dectin1-dependent differences in pathogenicity of SC5314 and ATCC18804 correlate with differences in chitin levels during infection *in vivo*. To test whether differences in chitin levels cause differential Dectin-1 dependence, we exploited our previous observation that *in vitro* treatment of *C. albicans* with Calcoflour White and CaCl_2_ induces high-chitin levels (Figure 7E) which can be maintained *in vivo* by treating mice with caspofungin [22]. Mirroring our *in vivo* findings (see Figure 6), we observed increased β-glucan exposure on high-chitin containing cells generated *in vitro* (Figure 7F). Using these *in vitro* treated fungal cells, we observed significantly increased resistance of Dectin-1^−/−^ mice following infection with high-chitin SC5314 (Figure 7G), compared to normal-chitin containing cells. In other words, the control of infection with high-chitin cells showed a reduced dependence on Dectin-1. Furthermore, using a model of peritoneal infection, we found that the inflammatory response to high-chitin SC5314 cells was Dectin-1 independent (Figure 7H). In contrast, the peritoneal inflammatory response to SC5314 cells with normal chitin levels required Dectin-1, as we had shown previously [6]. We conclude that variation in adaptability of individual strains of *C.albicans in vivo* results in substantial differences in cell wall architecture and exposure of PAMPs, particularly chitin, which significantly influences the innate recognition pathways utilized by the host during infection.

## Discussion

### Fungal strain-dependent requirement for Dectin-1 in protective anti-*Candida* immunity

Direct comparisons of previous studies on the role of Dectin-1 have been complicated by the use of different strains of *C. albicans* and different mouse genetic backgrounds [6], [11]. While mouse background can influence the immune response to *Candida* [23], [24], we have found that the discrepancies between these earlier studies on Dectin-1 stems from the use of different strains of *C. albicans*. Indeed, in complete agreement with the study by Iwakura [11], we could demonstrate that Dectin-1 was not involved in immunity to *C. albicans* ATCC18804, in terms of cytokine responses, fungal burdens or resistance to infection. In contrast, and consistent with our previous observations [6], [7], we found an absolute requirement for Dectin-1 for all these parameters in the control of infections with *C. albicans* SC5314. Notably, in both cases, these phenotypes were obtained irrespective of the mouse background tested. However, it is important to note that mouse strains do express different isoforms of Dectin-1 and that this can influence the type of immune response mounted [9], [13]. Furthermore, with other fungal pathogens, such as *Coccidioides*, these isoform differences can directly relate to resistance or susceptibility during systemic infection [25].

We also explored the possibility that Dectin-1 dependency correlated with the clade of *C. albicans*, but did not find any such association. This result is not particularly surprising, as the multilocus sequence typing used to assign strains to their various clades relies on sequence differences in just seven genes, none of which are related to fungal morphogenesis or cell wall architecture [15]. Furthermore, previous analyses have also not revealed any association between clade and fungal virulence or type of infection [26], [27].

### Differential adaptation of *C. albicans* strains *in vivo* and effect on immune recognition

Despite significant differences in the strain-specific Dectin-1 dependency *in vivo*, this phenotype could not be recapitulated *in vitro*. Unexpectedly, the composition of the cell walls of *in vitro* grown *C. albicans* ATCC18804 and SC5314 were nearly identical. Indeed, we found that macrophages could recognise and respond similarly to yeast and hyphal cells from both strains in a Dectin-1-dependent manner. We also studied the effect of hypoxia *in vitro*, which although understudied in this fungal species in the context of infection, is increasingly being appreciated to have a major influence on fungal adaptation and pathogenesis *in vivo*, including alterations in the cell wall [28], [29], [30]. In fact, in *Aspergillus*, we have recently shown that hypoxia enhances total and surface exposed β-glucan, leading to enhanced Dectin-1-dependent leukocyte inflammatory responses [18]. Consistent with these observations, we observed that hypoxia augments Dectin-1-dependent inflammatory responses to *C. albicans*, yet there was no difference in the leukocyte responses *in vitro* to each of the fungal strains tested. Interestingly, different strains of *C. albicans* have also been observed to have a variable dependency on TLR4 *in vivo* [31], [32], [33], but for this pattern recognition receptor these differences could be recapitulated *in vitro* and are thought to be related to differences in exposure of TLR4 ligands on the fungi [12]. Never-the-less, our observations highlight an inadequacy of studying *Candida*-host interactions *in vitro*.

We then examined the possibility that the dependency for Dectin-1 was related to the differential display of β-glucans *in vivo*, which occurs later during infection [17]. Consistent with this notion, we detected a substantial requirement for Dectin-1 at late stages of infection (day 9), with the appropriate fungal strain, whereas the control of all strains in the kidney was largely Dectin-1 independent at an earlier time point (day 3). Surprisingly, more β-glucan was exposed on the surface of the Dectin-1-independent strain (ATCC18804) *in vivo*, than the Dectin-1-dependent strain (SC5314) later during infection. However, altering the cell-wall architecture of ATCC18804 with caspofungin, converted this organism into a Dectin-1-dependent strain. Caspofungin inhibits the catalytic subunit of β-(1,3)-glucan synthase, which produces the β-1,3- glucan ligands that are recognised by Dectin-1 [34], [35]. Treatment with this echinocandin markedly alters the cell wall, inducing substantial changes in the β-glucan and chitin levels, which both become exposed at the cell surface [17], [20], [36]. Intriguingly, these alterations are thought to enhance Dectin-1 mediated recognition by unmasking cell-wall β-glucans [17]. However, ATCC18804 already possessed high levels of exposed β-glucans in untreated mice, hence other alterations in the cell wall were probably responsible for the conversion to a Dectin-1-dependent phenotype.

To identify factors that were contributing to the *in vivo* differences between these *C. albicans* strains, we analysed the expression of a number of selected cell-wall-associated genes in fungal cells isolated directly from infected kidneys. *C. albicans* is known to undergo substantial transcriptional reprogramming during infection, relating to changes in metabolism, stress responses, morphology and virulence [37], [38], [39], [40], [41]. Our analyses also revealed the dynamic regulation of cell wall- related genes upon infection, but we also observed significant differences in gene expression patterns between the two *C. albicans* strains during infection. This highlights strain-specific differences in host adaptation *in vivo*, and is consistent with previous observations [39], [40]. Remarkably, we also found striking differences in gene expression within the same strain, when comparing cells isolated from wild-type versus Dectin-1^−/−^ mice. For example, *CRH11* and *UTR2* encode GPI-anchored chitin-glucan cross-linking enzymes that were more highly expressed during infections of wild-type mice compared to the Dectin-1^−/−^ animals. These proteins are known to be antigenic [42] and the genes are up-regulated in response to caspofungin [43]. In *S. cerevisiae*, orthologues of these genes are included in a subset that is a signature for cell wall stress and a hallmark of activation of cell wall integrity pathways [44]. Thus, these data indicate that substantial changes in the *C. albicans* cell wall occur during infection, that there are strain-specific differences in host adaptation *in vivo*, and that this adaptation can be influenced by the immunological status of the host.

Of particular interest was the differential regulation of genes involved in regulating chitin biosynthesis, including *CHS3*, *PGA31* and *CHT2*. Notably, *CHS3* encodes Chs3p, a class IV chitin synthase which is responsible for the synthesis of short chitin-rodlets which make up the majority of the cell wall chitin in both yeast and hyphae [45]. These transcriptional analyses therefore suggested that there were differences in the amounts of chitin between these fungal strains *in vivo*; a conclusion we confirmed by demonstrating elevated chitin levels in ATCC18804 cells isolated from infected kidneys. Moreover, TEM analysis also demonstrated substantial differences in the architecture of their cell walls. We have previously shown that increased cell wall chitin in *C. albicans* can modulate inflammatory leukocyte responses *in vitro* [20]. Furthermore, we demonstrated here that high-chitin levels reduce the dependence on Dectin-1 *in vivo*, despite the presence of exposed β-glucans on the fungal cells. Thus variations in chitin content and differences in cell wall structure provide a rational explanation, at least in part, for the strain-specific differences in Dectin-1 dependency in our mouse models. While the mechanisms underlying these effects await further elucidation, our data indicate that detection of exposed β-glucans on fungal cells does not necessarily correlate with recognition by Dectin-1 *in vivo*.

### Implications for human infections

These observations have significant implications for our understanding of *C. albicans* infection in humans. We previously identified a polymorphism (Y238X) which could render individuals susceptible to infections with *C. albicans* and other fungi [5]. This polymorphism is common in many populations, yet its clinical penetrance is low, indicating that other factors are influencing susceptibility to infection [5]. Our data suggest that the effect of this polymorphism may only become apparent in individuals if they are infected with a Dectin-1-dependent strain of *C. albicans*. This link between host genotype and fungal strain needs to be explored in greater detail if we are to understand the contribution of polymorphisms of Dectin-1 and other PRRs to human disease susceptibility.

Our results also have implications for anti-fungal drug therapy. We have demonstrated, for example, that caspofungin treatment of ATCC18804 infections alters cell wall architecture and enables enhanced clearance *in vivo*. However, this treatment was not effective under conditions of Dectin-1 deficiency. Moreover, enhanced cell wall chitin levels can confer enhanced resistance to echinocandins [22], [36], [46], and reduce the dependency on Dectin-1 (Figure 4). These results underpin our previous *in vitro*-based observations suggesting that antifungal therapy should be targeted at more than one cell wall component [36].

In conclusion, our results provide substantial new insights into the interaction between *C. albicans* and its host and have significant implications for our understanding of anti-*Candida* immunity and drug treatment in humans.

## Materials and Methods

### Animals

Eight to 12-week old 129/Sv [6] and C57BL/6 (backcrossed for at least nine generations) *Clec7a* ^−/−^ (Dectin-1^−/−^) and wild-type (wt) mice were obtained from the specific pathogen free facilities of the University of Cape Town and University of Aberdeen. All animal experimentation was replicated at least twice using groups of 5–10 animals, unless otherwise stated. Mice were housed in groups in individually ventilated cages and provided with food and water *ad libitum*. Peritoneal thioglycollate-elicited macrophages were prepared as described previously [6]. All animal experimentation conformed to animal care and welfare protocols approved by the Universities of Aberdeen (project license numbers 60/4007 and 06/4135) and Cape Town (license number: 06/035) and in strict accordance with the guidelines for the usage of animal in laboratory research of the South African Association for Laboratory Animal Science and the UK home office.

### *C. albicans* strains and growth conditions

The *C. albicans* strains used in these experiments are listed in Table S1. *C. albicans* was maintained on YPD (Sigma-Aldrich) or Sabouraud (Oxoid) agar plates. For high chitin cells, cells were grown in YPD containing 0.2 M calcium chloride (CaCl_2_) and 100 µg/ml of Calcofluor White. For yeast cell preparation, cultures were incubated in Sabouraud broth (Oxoid) at 30°C for 24 h with shaking. To obtain hypoxic yeast cells, cultures were incubated under 1% O_2_ and 5% CO_2_ for the indicated times. Before use, the cells were washed in phosphate-buffered saline (PBS), and the cell density adjusted to the desired level with PBS. For inoculations, dosage was confirmed by viable cell counts on agar plates. To generate hyphae, a defined number of yeast cells were cultured at 37°C overnight in RPMI containing 10% heat-inactivated FCS. To obtain hypoxic cells, hyphae were cultured overnight under hypoxia, and washed as described above. Fungal cells were used either live or killed, by heat (100°C for 30 min) or UV-irradiation (6000 Joules), as indicated.

### *In vivo* inoculations and analyses

Mice were inoculated intravenously with the indicated doses of the various *C. albicans* strains in 100 µl sterile PBS. Mice were monitored daily and sacrificed at the indicated time points, or when judged to be moribund. In some experiments, mice were treated with 33 µg/kg caspofungin daily ip, starting one day post infection. Experiments were continued for a maximum of 21 days. Fungal burdens and cytokines in lysates from infected kidneys were determined as described previously [6]. Cytokines were normalised to lysate protein concentrations (BCA protein assay kit, Pierce).

Intraperitoneal infections were performed as described previously [6]. In brief, 1×10^5^ CFU in 100 µl PBS were injected i.p. into wt and Dectin-1^−/−^ mice and four hours post-infection, peritoneal inflammatory cells were harvested in PBS containing 5 mM EDTA. Cells were stained with CD11b-PEcy7 and Ly6G-APC (both from BD Biosciences) with neutrophils defined as CD11b^+^ Ly6G^hi^. Data was acquired on FACScalibur and analysed using FlowJo.

### Cell wall analysis

Cell wall mannan, β-glucan and chitin contents were determined by hydrolysis of these oligosaccharides and quantification by high-performance anion-exchange chromatography, as described previously [47]. To detect chitin, *ex vivo* isolated *C. albicans* cells were stained and quantified using Calcofluor White, as previously described [22]. TEM analysis was performed as previously described [36].

To detect exposed β-glucan, C57BL/6J mice were injected in the tail vein with 5.2×10^4^ CFU of either SC5314-GFP or ATCC18804-GFP. SC5314-GFP and ATCC18804-GFP strains were created by transformation with the pENO1-yEGFP3-NAT plasmid and verified by PCR as described previously [17]. After nine days, mice were sacrificed and the kidneys were harvested, homogenized, and processed as described [17]. Homogenates were stained with anti-β-glucan antibody (Biosupplies, Inc., Australia) at a concentration of 1.7 µg/ml, then stained with goat anti-mouse Cy3 antibody (Jackson Immunoresearch) at a concentration of 3.8 µg/ml. For soluble Dectin-1-Fc staining, homogenates were instead stained with Alexa647-labelled Dectin-1-Fc [48] at a concentration of 17 µg/ml and then with donkey anti-human IgG Cy3 antibody (Jackson Immunoresearch) at a concentration of 0.8 µg/ml. Cells were visualized by optical sectioning fluorescence microscopy using a Zeiss Axiovision Vivotome microscope (Carl Zeiss Microscopy, LLC). Live cells were identified based on characteristic EGFP fluorescence. Maximum projection images were quantified using Cellprofiler (www.cellprofiler.org) as described [17]. Briefly, EGFP fluorescence was used to manually define individual cell segments and average fluorescence intensity of β-glucan or Dectin-1-CRD fluorescence was measured for the whole cell segment. Cells labelled without primary antibody or Dectin-1-CRD were used as negative controls. *In vitro* grown cells were stained with soluble Dectin-1 at 5 µg/ml and then with anti-human IgG antibody (used at 1∶200) (Jackson Immunoresearch). Controls were stained with secondary antibody only.

### *C. albicans* gene expression analysis from normoxic and hypoxic *in vitro* cultures

*C. albicans* normoxic and hypoxic grown hyphae were re-suspended in Trizol reagent and chloroform to extract RNA. Tubes were centrifuged at 16,000× g for 15 min at 4°C. The clear upper layer was further extracted with an equal volume of 80% EtOH. Samples were applied to RNeasy spin columns (Qiagen RNA kit) following manufacturer's instructions. RNA was eluted with RNase free water.

RNA was DNase treated with DNA-free kit (Ambion) and reverse transcribed with QuantiTect reverse transcription kit (Qiagen, USA). Primers for all genes of interest were designed with PrimerQuest (IDT) and manufactured by IDT, USA. Sequences are: *FKS1*, Fwd-5′-TGATACTGGTAATCATAGACCAAAAA-3′, Rev- 5′-AACTCTGAATGGATTTGTAGAATAAGG-3′, *FKS2*, Fwd- 5′-ACTTGCTAGCAGTCGCCAAT-3′, Rev- 5′-ACCACCATGAGCGGTTAGAC-3′, *FKS3*, Fwd- 5′-ACCTCAATATTCAGCTTGGTGCCC-3′, Rev- 5′-GGACAACTCATTCGACTTGACCGT-3′, and *EFB1*, Fwd- 5′-CATTGATGGTACTACTGCCAC-3′, Rev- 5′-TTTACCGGCTGGCAAGTCTT-3′. All reactions were performed on BioRad MyIQ real-time PCR detection system with IQ SYBR green supermix (Bio-Rad, Hercules, CA). The ΔΔC_t_ method was used to assess changes in mRNA abundance, using *EBF1* as the housekeeping gene. Relative transcript abundances for *FKS1/2/3* are reported as means plus/minus SEM that are normalized to *EFB1* and presented as relative to the normoxic values for each strain.

### *C. albicans* gene expression analysis from infected tissue

*C.albicans*-enriched cortical shavings from two kidneys of the same animal were fixed in RNAlater according to manufacturer's instructions (Qiagen, Crawley, UK) and combined into a single sample for further processing. Approximately 3 mm^3^ of tissue were transferred to 600 µl QIAzol reagent (Qiagen, Crawley, UK), an equal volume of acid-washed glass beads added and the material homogenised using FastPrep-24 bead mill (10×20 sec bursts at 6.0 m/sec setting, with 4 min intervals on ice) (MP Biomedicals, Luton, UK). RNA extraction was carried out according to standard procedures. Nucleic acids were precipitated with RNA grade glycogen solution (Fermentas, Loughborough, UK). The final RNA pellet was suspended in 100 µl DEPC-treated water (DEPC-H_2_O) and further purified using NucleoSpin RNA Clean-up XS columns (Macherey-Nagel, Loughborough, UK). After repeated DNase I (Invitrogen, Paisley, UK) treatment the isolated RNA was assessed with NanoDrop ND-1000 spectrophotometer (Thermo Scientific, Loughborough, UK). Up to 40 ng isolated RNA was used in qRT-PCR reactions with *ACT1* gene primers to verify complete removal of genomic DNA (see below, and Table S2).

Total RNA (4.5 µg) was used in a cDNA synthesis reaction primed with a cocktail of all the qRT-PCR reverse primers (0.125 µM final primer concentration for each primer) (Table S2) and SuperScript II Reverse Transcriptase (Invitrogen, Paisley, UK). Single stranded cDNA was purified with QIAquick PCR Purification Kit (Qiagen, Crawley, UK), and eluted in 50 µl DEPC-H_2_O. qRT-PCR reactions were prepared in 10 µl volumes containing 2 µL of at least 10× diluted cDNA templates and the appropriate Universal Probes (Table S2) as per manufacturer's instructions, and run on a LightCycler 480 machine (Roche Applied Science, Burgess Hill, UK) using a Monocolour Hydrolysis Probe programme. All reactions were run at least in duplicate, with in-run *ACT1* gene standard. The relative transcript abundances normalised to *ACT1* were calculated based on the individually determined primer pair efficiencies with LightCycler 480 Software release 1.5.0.

### *In vitro* analyses

To measure binding and cytokine production, live *C. albicans* yeast cells were labelled with Rhodamine Green-X (Invitrogen) as described [6]. Labelled yeast cells were then added (MOI 5∶1 or 10∶1) to thioglycollate-elicited macrophages, which had been seeded the previous day at a density of 1–2.5*×*10^5^ cells/well in 24-well plates in RPMI media. After incubation for 30 min at 4°C, to allow particles to settle, followed by 30 min at 37°C, unbound particles were removed by washing. Cells were then cultured for a further 3 h at 37°C for analysis of proinflammatory cytokine production. After incubation, supernatants were stored at −80°C for subsequent TNF analysis. Cells were then lysed in 3% Triton X-100 and fluorescence measured using a Titer-Tek Fluoroskan II (Labsystems). TNF concentrations in the supernatants were measured by ELISA (OptEIA TNF kit; BD Pharmingen). Fluorescein isothiocyanate–labelled zymosan (Invitrogen) was used as a control in these experiments. In some experiments, as indicated, unlabelled UV-killed yeasts were used, the cells were not washed after addition of the particles, and TNF responses were assayed after overnight incubation at 37°C. The inflammatory response to hyphae was measured similarly, except that the thioglycollate-elicited peritoneal macrophages were directly added to live or heat-killed hyphae and supernatant samples taken after overnight incubation at 37°C. Isolation and stimulation of human PMBCs was performed as described previously [5].

### Statistics

All data were plotted using GraphPad Prism software. A two-tailed Student's t-test was used to analyse differences between two groups. The Mann-Whitney U test was used to determine statistical significance of differences between relative transcript abundances in each of the *in vivo* experimental groups. The reported values are arithmetical means of abundances normalised to *ACT1*, with the appropriate *p*-values from two-tailed t-test in pair-wise comparisons. Survival data were analyzed with the log rank test. Results were considered statistically significant with *p* values of less than 0.05.

## Supporting Information

Table S1 Details of *C. albicans* strains used in this study.
(DOCX)

Table S2 qRT-PCR primers and Universal Probes.
(DOCX)

## References

[1] Brown GD, Denning DW, Gow NA, Netea MG, White T (2012) Human fungal infections: The Hidden Killers. Sci Transl Med 4: 165rv113. 10.1126/scitranslmed.3004404 23253612

[2] Hernandez-Santos N, Gaffen SL (2012) Th17 cells in immunity to Candida albicans. Cell Host and Microbe 11: 425–435. 22607796 10.1016/j.chom.2012.04.008 PMC3358697

[3] Hardison SE, Brown GD (2012) C-type lectin receptors orchestrate antifungal immunity. Nat Immunol 13: 817–822. 22910394 10.1038/ni.2369 PMC3432564

[4] Iliev ID, Funari VA, Taylor KD, Nguyen Q, Reyes CN, et al (2012) Interactions between commensal fungi and the C-type lectin receptor Dectin-1 influence colitis. Science 336: 1314–1317. 22674328 10.1126/science.1221789 PMC3432565

[5] Ferwerda B, Ferwerda G, Plantinga TS, Willment JA, van Spriel AB, et al (2009) Human dectin-1 deficiency and mucocutaneous fungal infections. N Engl J Med 361: 1760–1767. 19864674 10.1056/NEJMoa0901053 PMC2773015

[6] Taylor PR, Tsoni SV, Willment JA, Dennehy KM, Rosas M, et al (2007) Dectin-1 is required for beta-glucan recognition and control of fungal infection. Nat Immunol 8: 31–38. 17159984 10.1038/ni1408 PMC1888731

[7] Hise AG, Tomalka J, Ganesan S, Patel K, Hall BA, et al (2009) An essential role for the NLRP3 inflammasome in host defense against the human fungal pathogen Candida albicans. Cell Host Microbe 5: 487–497. 19454352 10.1016/j.chom.2009.05.002 PMC2824856

[8] Gales A, Conduche A, Bernad J, Lefevre L, Olagnier D, et al (2010) PPARgamma controls dectin-1 expression required for host antifungal defense against Candida albicans. PLoS Pathog 6: e1000714. 20062524 10.1371/journal.ppat.1000714 PMC2795865

[9] Carvalho A, Giovannini G, De Luca A, D'Angelo C, Casagrande A, et al (2012) Dectin-1 isoforms contribute to distinct Th1/Th17 cell activation in mucosal candidiasis. Cell Mol Immunol 9: 276–286. 22543832 10.1038/cmi.2012.1 PMC4012853

[10] Werner JL, Metz AE, Horn D, Schoeb TR, Hewitt MM, et al (2009) Requisite role for the dectin-1 beta-glucan receptor in pulmonary defense against Aspergillus fumigatus. J Immunol 182: 4938–4946. 19342673 10.4049/jimmunol.0804250 PMC3434356

[11] Saijo S, Fujikado N, Furuta T, Chung SH, Kotaki H, et al (2007) Dectin-1 is required for host defense against Pneumocystis carinii but not against Candida albicans. Nat Immunol 8: 39–46. 17159982 10.1038/ni1425

[12] Netea MG, Gow NA, Joosten LA, Verschueren I, van der Meer JW, et al (2010) Variable recognition of Candida albicans strains by TLR4 and lectin recognition receptors. Med Mycol 48: 897–903. 20166865 10.3109/13693781003621575

[13] Heinsbroek SE, taylor PR, Rosas M, Willment JA, Williams DL, et al (2006) Expression of functionally different Dectin-1 isoforms by murine macrophages. J Immunol 176: 5513–5518. 16622020 10.4049/jimmunol.176.9.5513

[14] Whiley RA, Cruchley AT, Gore C, Hagi-Pavli E (2012) Candida albicans strain-dependent modulation of pro-inflammatory cytokine release by in vitro oral and vaginal mucosal models. Cytokine 57: 89–97. 22129624 10.1016/j.cyto.2011.10.017

[15] Odds FC, Bougnoux ME, Shaw DJ, Bain JM, Davidson AD, et al (2007) Molecular phylogenetics of Candida albicans. Eukaryot Cell 6: 1041–1052. 17416899 10.1128/EC.00041-07 PMC1951527

[16] Gantner BN, Simmons RM, Underhill DM (2005) Dectin-1 mediates macrophage recognition of Candida albicans yeast but not filaments. Embo J 24: 1277–1286. 15729357 10.1038/sj.emboj.7600594 PMC556398

[17] Wheeler RT, Kombe D, Agarwala SD, Fink GR (2008) Dynamic, morphotype-specific Candida albicans beta-glucan exposure during infection and drug treatment. PLoS Pathog 4: e1000227. 19057660 10.1371/journal.ppat.1000227 PMC2587227

[18] Shepardson KM, Ngo LY, Aimanianda V, Latge JP, Barker BM, et al (2012) Hypoxia enhances innate immune activation to Aspergillus fumigatus through cell wall modulation. Microbes Infect In Press. 10.1016/j.micinf.2012.11.010 PMC3723392 23220005

[19] Netea MG, Brown GD, Kullberg BJ, Gow NA (2008) An integrated model of the recognition of Candida albicans by the innate immune system. Nat Rev Microbiol 6: 67–78. 18079743 10.1038/nrmicro1815

[20] Mora-Montes HM, Netea MG, Ferwerda G, Lenardon MD, Brown GD, et al (2011) Recognition and blocking of innate immunity cells by Candida albicans chitin. Infect Immun 79: 1961–1970. 21357722 10.1128/IAI.01282-10 PMC3088140

[21] Plaine A, Walker L, Da Costa G, Mora-Montes HM, McKinnon A, et al (2008) Functional analysis of Candida albicans GPI-anchored proteins: roles in cell wall integrity and caspofungin sensitivity. Fungal Genet Biol 45: 1404–1414. 18765290 10.1016/j.fgb.2008.08.003 PMC2649418

[22] Lee KK, Maccallum DM, Jacobsen MD, Walker LA, Odds FC, et al (2012) Elevated cell wall chitin in Candida albicans confers echinocandin resistance in vivo. Antimicrob Agents Chemother 56: 208–217. 21986821 10.1128/AAC.00683-11 PMC3256049

[23] Hector RF, Domer JE, Carrow EW (1982) Immune responses to Candida albicans in genetically distinct mice. Infect Immun 38: 1020–1028. 6759403 10.1128/iai.38.3.1020-1028.1982 PMC347851

[24] Ashman RB, Fulurija A, Papadimitriou JM (1996) Strain-dependent differences in host response to Candida albicans infection in mice are related to organ susceptibility and infectious load. Infect Immun 64: 1866–1869. 8613406 10.1128/iai.64.5.1866-1869.1996 PMC174007

[25] Del Pilar Jimenez AM, Viriyakosol S, Walls L, Datta SK, Kirkland T, et al (2008) Susceptibility to Coccidioides species in C57BL/6 mice is associated with expression of a truncated splice variant of Dectin-1 (Clec7a). Genes Immun 9: 338–348. 18418396 10.1038/gene.2008.23 PMC3681288

[26] MacCallum DM, Castillo L, Nather K, Munro CA, Brown AJ, et al (2009) Property differences among the four major Candida albicans strain clades. Eukaryot Cell 8: 373–387. 19151328 10.1128/EC.00387-08 PMC2653250

[27] Odds FC (2010) Molecular phylogenetics and epidemiology of Candida albicans. Future Microbiol 5: 67–79. 20020830 10.2217/fmb.09.113

[28] Synnott JM, Guida A, Mulhern-Haughey S, Higgins DG, Butler G (2010) Regulation of the hypoxic response in Candida albicans. Eukaryot Cell 9: 1734–1746. 20870877 10.1128/EC.00159-10 PMC2976306

[29] Grahl N, Shepardson KM, Chung D, Cramer RA (2012) Hypoxia and fungal pathogenesis: to air or not to air? Eukaryot Cell 11: 560–570. 22447924 10.1128/EC.00031-12 PMC3346435

[30] Sosinska GJ, de Groot PW, Teixeira de Mattos MJ, Dekker HL, de Koster CG, et al (2008) Hypoxic conditions and iron restriction affect the cell-wall proteome of Candida albicans grown under vagina-simulative conditions. Microbiology 154: 510–520. 18227255 10.1099/mic.0.2007/012617-0

[31] Murciano C, Villamon E, Gozalbo D, Roig P, O'Connor JE, et al (2006) Toll-like receptor 4 defective mice carrying point or null mutations do not show increased susceptibility to Candida albicans in a model of hematogenously disseminated infection. Med Mycol 44: 149–157. 16519018 10.1080/13693780500294733

[32] Netea MG, Van Der Graaf CA, Vonk AG, Verschueren I, Van Der Meer JW, et al (2002) The role of toll-like receptor (TLR) 2 and TLR4 in the host defense against disseminated candidiasis. J Infect Dis 185: 1483–1489. 11992285 10.1086/340511

[33] Bellocchio S, Montagnoli C, Bozza S, Gaziano R, Rossi G, et al (2004) The contribution of the toll-like/IL-1 receptor superfamily to innate and adaptive immunity to fungal pathogens in vivo. J Immunol 172: 3059–3069. 14978111 10.4049/jimmunol.172.5.3059

[34] Adams EL, Rice PJ, Graves B, Ensley HE, Yu H, et al (2008) Differential high affinity interaction of Dectin-1 with natural or synthetic glucans is dependent upon primary structure and is influenced by polymer chain length and side chain branching. J Pharmacol Exp Ther 325: 115–123. 18171906 10.1124/jpet.107.133124

[35] Palma AS, Feizi T, Zhang Y, Stoll MS, Lawson AM, et al (2006) Ligands for the beta-glucan receptor, Dectin-1, assigned using “designer” microarrays of oligosaccharide probes (neoglycolipids) generated from glucan polysaccharides. J Biol Chem 281: 5771–5779. 16371356 10.1074/jbc.M511461200

[36] Walker LA, Munro CA, de Bruijn I, Lenardon MD, McKinnon A, et al (2008) Stimulation of chitin synthesis rescues Candida albicans from echinocandins. PLoS Pathog 4: e1000040. 18389063 10.1371/journal.ppat.1000040 PMC2271054

[37] Staib P, Kretschmar M, Nichterlein T, Hof H, Morschhauser J (2000) Differential activation of a Candida albicans virulence gene family during infection. Proc Natl Acad Sci U S A 97: 6102–6107. 10811913 10.1073/pnas.110031497 PMC18565

[38] Lorenz MC, Bender JA, Fink GR (2004) Transcriptional response of Candida albicans upon internalization by macrophages. Eukaryot Cell 3: 1076–1087. 15470236 10.1128/EC.3.5.1076-1087.2004 PMC522606

[39] Thewes S, Kretschmar M, Park H, Schaller M, Filler SG, et al (2007) In vivo and ex vivo comparative transcriptional profiling of invasive and non-invasive Candida albicans isolates identifies genes associated with tissue invasion. Mol Microbiol 63: 1606–1628. 17367383 10.1111/j.1365-2958.2007.05614.x

[40] Walker LA, Maccallum DM, Bertram G, Gow NA, Odds FC, et al (2009) Genome-wide analysis of Candida albicans gene expression patterns during infection of the mammalian kidney. Fungal Genet Biol 46: 210–219. 19032986 10.1016/j.fgb.2008.10.012 PMC2698078

[41] Andes D, Lepak A, Pitula A, Marchillo K, Clark J (2005) A simple approach for estimating gene expression in Candida albicans directly from a systemic infection site. J Infect Dis 192: 893–900. 16088840 10.1086/432104

[42] Arroyo J, Sarfati J, Baixench MT, Ragni E, Guillen M, et al (2007) The GPI-anchored Gas and Crh families are fungal antigens. Yeast 24: 289–296. 17397107 10.1002/yea.1480

[43] Liu TT, Lee RE, Barker KS, Wei L, Homayouni R, et al (2005) Genome-wide expression profiling of the response to azole, polyene, echinocandin, and pyrimidine antifungal agents in Candida albicans. Antimicrob Agents Chemother 49: 2226–2236. 15917516 10.1128/AAC.49.6.2226-2236.2005 PMC1140538

[44] Garcia R, Bermejo C, Grau C, Perez R, Rodriguez-Pena JM, et al (2004) The global transcriptional response to transient cell wall damage in Saccharomyces cerevisiae and its regulation by the cell integrity signaling pathway. J Biol Chem 279: 15183–15195. 14739279 10.1074/jbc.M312954200

[45] Lenardon MD, Whitton RK, Munro CA, Marshall D, Gow NA (2007) Individual chitin synthase enzymes synthesize microfibrils of differing structure at specific locations in the Candida albicans cell wall. Mol Microbiol 66: 1164–1173. 17971081 10.1111/j.1365-2958.2007.05990.x PMC2780561

[46] Drakulovski P, Dunyach C, Bertout S, Reynes J, Mallie M (2011) A Candida albicans strain with high MIC for caspofungin and no FKS1 mutations exhibits a high chitin content and mutations in two chitinase genes. Med Mycol 49: 467–474. 21108572 10.3109/13693786.2010.538732

[47] Mora-Montes HM, Bates S, Netea MG, Diaz-Jimenez DF, Lopez-Romero E, et al (2007) Endoplasmic reticulum alpha-glycosidases of Candida albicans are required for N glycosylation, cell wall integrity, and normal host-fungus interaction. Eukaryot Cell 6: 2184–2193. 17933909 10.1128/EC.00350-07 PMC2168260

[48] Graham LM, Tsoni SV, Willment JA, Williams DL, Taylor PR, et al (2006) Soluble Dectin-1 as a tool to detect beta-glucans. J Immunol Methods 314: 164–169. 16844139 10.1016/j.jim.2006.05.013

