# Correction: Differential Adaptation of *Candida albicans* In Vivo Modulates Immune Recognition by Dectin-1

**DOI:** 10.1371/annotation/7821bda1-dde3-4e72-b688-447b6bca20ea

**Published:** 2013-10-30

**Authors:** Mohlopheni J. Marakalala, Simon Vautier, Joanna Potrykus, Louise A. Walker, Kelly M. Shepardson, Alex Hopke, Hector M. Mora-Montes, Ann Kerrigan, Mihai G. Netea, Graeme I. Murray, Donna M. MacCallum, Robert Wheeler, Carol A. Munro, Neil A. R. Gow, Robert A. Cramer, Alistair J. P. Brown, Gordon D. Brown

Keunsook K. Lee has been added to this article as an author. The updated author order is as follows:

Mohlopheni J. Marakalala, Simon Vautier, Joanna Potrykus, Louise A. Walker, Kelly M. Shepardson, Alex Hopke, Hector M. Mora-Montes, Keunsook K. Lee, Ann Kerrigan, Mihai G. Netea, Graeme I. Murray, Donna M. MacCallum, Robert Wheeler, Carol A. Munro, Neil A. R. Gow, Robert A. Cramer, Alistair J. P. Brown, Gordon D. Brown

The following is the affiliation for Keunsook K. Lee:

2 Aberdeen Fungal Group, University of Aberdeen, Institute of Medical Sciences, Foresterhill, Aberdeen, United Kingdom

Author Keunsook K. Lee's has been added to the following section in the author contributions list:

Performed the experiments: MJM SV JP LAW KMS AH HMM AK MGN KL 

