# Supplementary material for: Differential Adaptation of Candida albicans In Vivo Modulates Immune Recognition by Dectin-1
Source: PLoS Pathog. 2013 Apr 18;9(4):e1003315. doi: 10.1371/journal.ppat.1003315 (PMC3630191; doi:10.1371/journal.ppat.1003315)
Supplement: Table S1 — Details of C. albicans strains used in this study. (DOCX) [file ppat.1003315.s001.docx]

**Table S1:** Details of *C. albicans* strains used in this study^a^

| **Strain Designation** | **DST** | **Clade** | **sample or**  **anatomical source^b^** | **virulence^c^** |
| --- | --- | --- | --- | --- |
| SC5314 | 52 | 1 | generalized infection | 5*10^3^ |
| ATCC18804 | 1030 | 5 | skin - interdigital mycosis | 5*10^5^ |
| AM2003-016 | 123 | 1 | oral | 5*10^4^ |
| FJ9 | 102 | 1 | throat | 5*10^4^ |
| FC22 | 155 | 2 | vulva | 5*10^4^ |
| GP-1 | 344 | 3 | vagina | 5*10^3^ |
| s20175.016 | 124 | 4 | blood | 5*10^3^ |
| AM2005/0425 | 728 | 5 | blood | 5*10^4^ |
| B600142/00/1 | 365 | 8 | pigeon | 5*10^4^ |
| F173 | 538 | 11 | nail | 5*10^4^ |
| AM2003/0025 | 182 | 13 | vagina | 5*10^4^ |
| 81/078 | 147 | 5 | vagina | 5*10^4^ |
| B30/97/1 | 1021 | 10 | partridge | 5*10^4^ |

^a^ Odds FC, Bougnoux ME, Shaw DJ, Bain JM, Davidson AD, et al. (2007) Molecular phylogenetics of *Candida albicans*. Eukaryot Cell 6: 1041-1052.

^b^Human samples, unless otherwise indicated

^c^CFUs required for 50-75% mortality in our systemic model
