# Supplementary material for: Differential Adaptation of Candida albicans In Vivo Modulates Immune Recognition by Dectin-1
Source: PLoS Pathog. 2013 Apr 18;9(4):e1003315. doi: 10.1371/journal.ppat.1003315 (PMC3630191; doi:10.1371/journal.ppat.1003315)
Supplement: Table S2 — qRT-PCR primers and Universal Probes. (DOCX) [file ppat.1003315.s002.docx]

**Table S2.** qRT-PCR primers and Universal Probes^1^

| **GENE** | **ORF** | **primer name** | **5' TO 3'** |  | **length** | **Tm**  **(⁰C)** | **Universal Probe**^1^ |
| --- | --- | --- | --- | --- | --- | --- | --- |
| **ACT1** | orf19.5007 | ACT1_LEFT | AACCACCGGTATTGTTTTGG | | 20 | 59 | 9 |
|  |  | ACT1_RIGHT | GCGTAAATTGGAACAACGTG | | 20 | 59 | 9 |
| **ACE2** | orf19.6124 | ACE2.F | CAACCATTACACAAACAAATCAAAG | | 25 | 59 | 9 |
|  |  | ACE2.R | TCACCTGGTGGTAACAATGAAG | | 22 | 59 | 9 |
| **ALS1** | orf19.5741 | ALS1_LEFT | GACAACAAAAGCGGTTCTCAT | | 21 | 59 | 89 |
|  |  | ALS1_RIGHT | TTTCACCCATACTTGGTTTCAA | | 22 | 59 | 89 |
| **ALS3** | orf19.1816 | ALS3_LEFT | CTGCTGAATCAACCAGTGTCA | | 21 | 59 | 77 |
|  |  | ALS3_RIGHT | GATGGAGATGAAGTTGAAGTTGC | | 23 | 60 | 77 |
| **BGL2** | orf19.4565 | BGL2.F | TGAAAATGCTGCTGATCAATG | | 21 | 59 | 99 |
|  |  | BGL2.R | GCAACATTAATACCCCAAGCTC | | 22 | 59 | 99 |
| **CHS3** | orf19.4937 | CHS3.F_3 | GGCAGAATTCTATGAGATTGTATTGA | | 26 | 59 | 89 |
|  |  | CHS3.R_3 | GTTAAAGAATCAGGGAAAACTTTTGT | | 26 | 59 | 89 |
| **CHT2** | orf19.3895 | CHT2.F_2 | TCCCAACTGGTTCTAACGAAA | | 21 | 59 | 87 |
|  |  | CHT2.R_2 | TTGAAATGGCAAGTTTGGTG | | 20 | 59 | 87 |
| **CHT3** | orf19.7586 | CHT3.F_2 | TGTTAAGAAAGCTTCACCAGGAG | | 23 | 59 | 143 |
|  |  | CHT3.R_2 | TTGTTGTTGAAGTAGTAGTGGTGGT | | 25 | 59 | 143 |
| **CRH11** | orf19.2706 | CRH11.F | CTGGTACTACTAGTGGGTCTGCAA | | 24 | 59 | 78 |
|  |  | CRH11.R | TGGAGGCAGTACTTGAAGCAG | | 21 | 60 | 78 |
| **CSA1** | orf19.7114 | CSA1_LEFT | TGGCTGCTGTACAATCATCTG | | 21 | 59 | 132 |
|  |  | CSA1_RIGHT | TGCCTCAGACGACTTTTGG | | 19 | 59 | 132 |
| **CSA2** | orf19.3117 | CSA2_LEFT | TTTTCTACTATTTTAGCCATTCCATTT | | 27 | 59 | 132 |
|  |  | CSA2_RIGHT | GCTGGGGCAGCAGTAACA | | 18 | 60 | 132 |
| **FKS1** | orf19.2929 | FKS1.F_2 | GAATGAGGACGAGGAACCAA | | 20 | 60 | 13 |
|  |  | FKS1.R_2 | TCTAACATTTCACAATGACCATCA | | 24 | 59 | 13 |
| **FTR1** | orf19.7219 | FTR1_LEFT | TCAATGCCATTTTGGGATG | | 19 | 60 | 53 |
|  |  | FTR1_RIGHT | AAATGTTGTAGGAAATGACAGAACC | | 25 | 59 | 53 |
| **GFA1** | orf19.1618 | GFA1.F | TGGTGTTGCCTCAACTAAAGC | | 21 | 60 | 9 |
|  |  | GFA1.R | TTCCCTTTCTGGAAATAGAATCA | | 23 | 59 | 9 |
| **HMX1** | orf19.6073 | HMX1_LEFT | TGACGGAAGAACAAAAGTTGG | | 21 | 60 | 5 |
|  |  | HMX1_RIGHT | CCCGAAAGTTTGTCCATGTT | | 20 | 59 | 5 |
| **HWP1** | orf19.1321 | HWP1_LEFT | GTCCATTGACTGAAACTACTCCAG | | 24 | 59 | 19 |
|  |  | HWP1_RIGHT | TGGATACTGTACCAGTTGGTGTTT | | 24 | 59 | 19 |
| **KRE6** | orf19.7363 | KRE6.F | AATGACCCTGAAACGGGTTA | | 20 | 59 | 9 |
|  |  | KRE6.R | TTGTGAATAAACCGTTAATGTTGG | | 24 | 60 | 9 |
| **KRE9** | orf19.5861 | KRE9.F_2 | CCACGTTTCAAATTGACTGG | | 20 | 59 | 93 |
|  |  | KRE9.R_2 | CACTGATCCTGTTTCGGTGA | | 20 | 59 | 93 |
| **MKC1** | orf19.7523 | MKC1.F_2 | CGACGAAAATGCTGGGTTTA | | 20 | 60 | 145 |
|  |  | MKC1.R_2 | TCTGGTGCCCTGTACCATCT | | 20 | 60 | 145 |
| **PGA31** | orf19.5302 | PGA31.F_3 | CCAAAGATTCATATGCCGTTG | | 21 | 60 | 9 |
|  |  | PGA31.R_3 | GCAACAAGTTTGATTGGAATAGC | | 23 | 60 | 9 |
| **PGA7** | orf19.5635 | PGA7.F_2 | TTGGATGATGCCTGCTTCTA | | 20 | 59 | 86 |
|  |  | PGA7.R_2 | TTGTAGTAACTTCCTTAGCATCTCCA | | 26 | 59 | 86 |
| **PHR1** | orf19.3829 | PHR1_LEFT | TCTGGTGGAAGCTCCAAATC | | 20 | 60 | 6 |
|  |  | PHR1_RIGHT | GGTGCTGCTGCTTGATGA | | 18 | 59 | 6 |
| **PHR2** | orf19.6081 | PHR2_LEFT | GAAGTGGCTACTCTTTTTGGTGA | | 23 | 59 | 109 |
|  |  | PHR2_RIGHT | ACATGTAAACAATACCACCAGACC | | 24 | 59 | 109 |
| **SAP2** | orf19.3708 | SAP2.F_2 | TGATAATGTCGATGTTCTTGTGG | | 23 | 59 | 157 |
|  |  | SAP2.R_2 | AGCTTTAATGATTTGATCAGCAAG | | 24 | 59 | 157 |
| **SAP5** | orf19.5585 | SAP5_LEFT | AGGTAAGTTGTATAAAGATACCGTTGG | | 27 | 59 | 119 |
|  |  | SAP5_RIGHT | CCTTACGAGCACTAGTAGACCAAA | | 24 | 59 | 119 |
| **SAP9** | orf19.6928 | SAP9.F | GGCGCTTACGTAGTCAACTGT | | 21 | 59 | 67 |
|  |  | SAP9.R | CCACCAAATTCTATGTCGACTG | | 22 | 59 | 67 |
| **SOD5** | orf19.2060 | SOD5_LEFT | ACAATGGAACCGTTAGAGCTG | | 21 | 59 | 132 |
|  |  | SOD5_RIGHT | TTTCCAGCCAAATCACCAAC | | 20 | 60 | 132 |
| **UTR2** | orf19.1671 | UTR2.F | CAAGATTCAAAATCTACTGATTCTGGT | | 27 | 60 | 38 |
|  |  | UTR2.R | TGAATTGGCAACACCTTGAG | | 20 | 59 | 38 |
| **YWP1** | orf19.3618 | YWP1_LEFT | CCATTAAGTTCTTATGAAACCGTTG | | 25 | 60 | 9 |
|  |  | YWP1_RIGHT | TCTTGACATTTGTTTTCACTACATGA | | 26 | 59 | 9 |

^1^ All primers were designed using the Universal Probe Library Assay Design Centre available on line from Roche Applied Science (http://www.roche-applied-science.com/sis/rtpcr/upl/index.jsp?id=UP030000).
